# Effectiveness of mixed reality-based rehabilitation on hands and fingers by individual finger-movement tracking in patients with stroke

**DOI:** 10.1186/s12984-024-01418-6

**Published:** 2024-08-10

**Authors:** Yeajin Ham, Dong-Seok Yang, Younggeun Choi, Joon-Ho Shin

**Affiliations:** 1 Department of Rehabilitation Medicine, National Rehabilitation Center, Ministry of Health and Welfare, 58, Samgaksan-ro, Gangbuk-gu, Seoul, 01022 Republic of Korea; 2 Business Growth Support Center, Seongnam, 13449 Korea; 3 Department of Computer Engineering, Dankook University, Yongin-si, 16890 Gyeonggi-do Korea

**Keywords:** Mixed reality, Fingers, Stroke rehabilitation, Equipment and supplies, Wearable device, Range of motion

## Abstract

**Background:**

Mixed reality (MR) is helpful in hand training for patients with stroke, allowing them to fully submerge in a virtual space while interacting with real objects. The recognition of individual finger movements is required for MR rehabilitation. This study aimed to assess the effectiveness of updated MR-board 2, adding finger training for patients with stroke.

**Methods:**

Twenty-one participants with hemiplegic stroke (10 with left hemiplegia and 11 with right hemiplegia; nine female patients; 56.7 ± 14.2 years of age; and onset of stroke 32.7 ± 34.8 months) participated in this study. MR-board 2 comprised a board plate, a depth camera, plastic-shaped objects, a monitor, a palm-worn camera, and seven gamified training programs. All participants performed 20 self-training sessions involving 30-min training using MR-board 2. The outcome measurements for upper extremity function were the Fugl–Meyer assessment (FMA) upper extremity score, repeated number of finger flexion and extension (Repeat-FE), the thumb opposition test (TOT), Box and Block Test score (BBT), Wolf Motor Function Test score (WMFT), and Stroke Impact Scale (SIS). One-way repeated measures analysis of variance and the post hoc test were applied for the measurements. MR-board 2 recorded the fingers’ active range of motion (AROM) and Dunnett’s test was used for pairwise comparisons.

**Results:**

Except for the FMA-proximal score (*p* = 0.617) and TOT (*p* = 0.005), other FMA scores, BBT score, Repeat-FE, WMFT score, and SIS stroke recovery improved significantly (*p* < 0.001) during MR-board 2 training and were maintained until follow-up. All AROM values of the finger joints changed significantly during training (*p* < 0.001).

**Conclusions:**

MR-board 2 self-training, which includes natural interactions between humans and computers using a tangible user interface and real-time tracking of the fingers, improved upper limb function across impairment, activity, and participation. MR-board 2 could be used as a self-training tool for patients with stroke, improving their quality of life.

*Trial registration number:* This study was registered with the Clinical Research Information Service (CRIS: KCT0004167).

**Supplementary Information:**

The online version contains supplementary material available at 10.1186/s12984-024-01418-6.

## Background

Stroke is a prevalent, severe, and incapacitating worldwide health issue, and a key component of stroke care is rehabilitation [1]. Continuous and sufficient rehabilitation is required to elicit functional improvement [2]. Several augmented and virtual reality applications have been implemented to enhance rehabilitation [3]. Mixed reality (MR), which blends virtual reality and physical things, allows participants to fully submerge themselves into a virtual space by interacting with real objects, thereby maintaining their sense of reality. Previous studies have demonstrated the feasibility of MR-based rehabilitation (MRR) specifically for upper limb rehabilitation among participants with stroke [4, 5]. The real physical objects of MRR play the role of tangible user interfaces, enabling more engagement, active participation, and effective learning [6, 7]. MRR could be useful for hand rehabilitation because the physical interfaces provide a haptic sense to the contacting hand, which is a gate for the interaction of the body with objects [8].

Finger individuation can be impaired even by small or lacunar lesions resulting from a stroke [9]. This impaired individuation affects a range of activities, such as typing, grasping, and transporting of objects [10]. Reduced finger strength and impaired finger individuation are two motor deficits affecting hand function following stroke [11]. The potential benefits of the MRR can be achieved through complex hand movement that require individual finger movements. Colomer et al. presented an MRR program that included finger tapping, pincer grasping, and mass grasping [5]. However, recognizing individual finger movements is challenging in previously introduced MR systems because they are only sensed using a depth-perception camera, not collecting kinematic data [5, 8]. Capturing the entire finger movement is particularly difficult for stroke participants because they commonly experience spasticity, dystonia, or deformities, which impede adequate movement perception from the camera [12, 13]. Various types of sensors, including wearable and flexible sensors and inertial measurement unit (IMU) sensors, have been used for fingers [14–16]. However, sensing using an IMU sensor is affected by attachment location, and wearable-type sensors are difficult to wear by participants with stroke.

To address these issues, we updated the MRR system (MR-board 2) by adding a palm camera (TapSix) and specific training programs for fingers [17]. We originally developed an MR board for hand rehabilitation and demonstrated the feasibility of the MR board as a self-training tool for the upper extremity in patients with stroke [8]. The MR board provided interventions regarding gross hand movements only and did not include individual finger training (FT). The newly developed MR-board 2 can provide finger-relevant training, allowing for more hierarchical training according to the participants’ capabilities and goals. When participants could not train their fingers at the initial stage, they received gross hand training, such as grasping, releasing, and object manipulation. If they regain finger function, they can move on to individual FT.

Therefore, we hypothesized that MR-board 2 could benefit upper-limb self-rehabilitation, especially for hand rehabilitation, including FT and capturing entire finger movements. This study aimed to apply MR-board 2 to participants with stroke as a tool for self-rehabilitation and explore its effectiveness across every domain (impairment, limitation, and restriction) of the International Classification of Functioning, Disability, and Health (ICF) [18]. We also recorded and analyzed each joint involved in the entire finger movement during FT.

## Methods

The present study was performed at a single rehabilitation hospital using a pre–post design. The institutional review board of our rehabilitation hospital approved this study (NRC-2018-04-026), and all participants provided written informed consent before enrollment.

### Participants

The inclusion criteria were as follows: (1) age > 19 years; (2) unilateral upper limb functional deficits secondary to first-ever hemispheric stroke as identified from the medical record; (3) participants with chronic stroke, as defined by stroke duration > 6 months; (4) Participants who did not receive any other physical rehabilitation interventions (services provided by any type of healthcare professional from a medical center) other than the MR-board intervention during the present study. We did not control for other exercises or interventions not provided by medical centers (e.g., participating in self-training or group exercises from community care centers); (5) Brunnstrom's motor recovery stage in the affected arm and hand ≥ 4 [19]; (6) the Medical Research Council scale of muscle strength for wrist flexion/extension, forearm pronation/supination, and finger flexion/extension strength ≥ 3 [20]; and (7) cognitive ability to understand and follow instructions (mini-mental state examination score ≥ 24) [21]. The exclusion criteria were as follows: (1) stroke of bilateral brain lesions; (2) any neurological disorders other than stroke; (3) Modified Ashworth Scale (MAS) score of upper limb spasticity ≥ 2 [22]; (4) predisposing severe pain in the upper limb that could impede training; (5) any severe medical condition; and (6) inability to follow instructions because of cognitive impairment or severe aphasia.

Twenty-one participants were included in this study. The demographic information of the selected participants is presented in Table 1.

**Table 1:** Demographic and clinical characteristics of study participants

Variable	Total participants (n = 21)	Brunnstrom stage 4 (n = 10)	Brunnstrom stage 5 or 6 (n = 11)
	Mean ± standard error or n (%)		
Age (19–84)	56.7 ± 14.2 (55^†^)	61.1 ± 15.5 (54^†^)	52.7 ± 12.2 (55^†^)
Sex (female)	9 (42.9%)	4 (40%)	5 (45.5%)
Time from the stroke (months)	32.7 ± 34.8	28.6 ± 22.8	36.5 ± 43.8
Affected side (left)	10 (47.6%)	5 (50%)	5 (45.5%)

^†^Median of age

### Apparatus

#### Instrument description

**Fig. 1:**
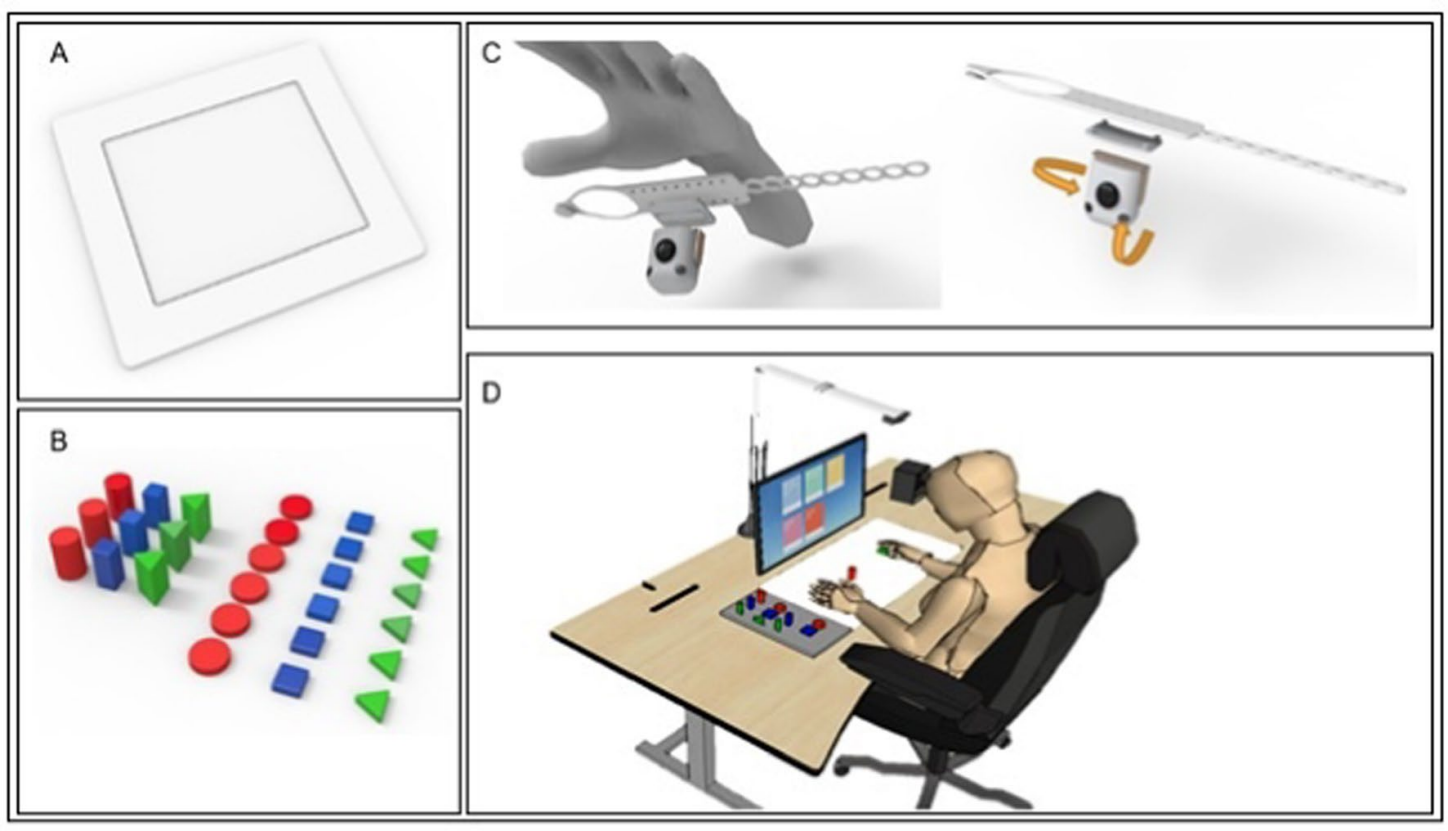
Description of MR-board-2 components. **A** Main MR board. **B** Six objects with different shapes and sizes. **C** TapSix system worn on the palm to capture finger motion. **D** Schematic illustration of training using the MR-board 2. MR, mixed reality

The original version of the MR board comprised a board plate, a depth camera, plastic-shaped objects, and a monitor [8]. The board surface can be applied differently with multiple textures, providing various haptic senses (rough or soft) to the participants’ fingertips. MR-board 2 was updated by adding a palm-worn camera (TapSix system) to record individual finger movements and a finger-specific training program [17]. The TapSix system was placed on the palm of the participants, specifically in the hypothenar area, instead of making them wear a camera on the wrist, which requires a wide range of motion (RoM), allowing a stable angle of view without missing finger images owing to the occlusion of the camera. The primary components of the TapSix are a Raspberry Pi Zero with a Broadcom BCM2385 processor, an inexpensive camera sensor (OV5647, Omnibus), and a Bluetooth module with support for human-interface devices (FB155BC, Firmtech). A silicone band fixes the camera without occlusion of finger movement. The TapSix battery lasted for 3 h with 580 mA of current and 1700 mAh of capacity. Through image processing, TapSix identified the fingertip apart from the surrounding environment on various surfaces and determined finger tapping on the tactile surface by computing the shortest distance between the fingertip position and the surface edge. Hand-pose estimation technology, which can extract the movement of every finger joint, was used to analyze the participants' hand movements. In this system, hand position does not affect tracking and detection through calibration, and hand orientation has no effect because it was systematically limited. The detailed components of MR-board 2 and schematic illustration of training are illustrated in Fig. 1.

#### Contents of training programs

MR-board 2 contains seven gamified training programs, which are categorized into 1) training with a bare hand (virtual hand training [ViHT]), 2) training using tangible objects (tangible hand training [TaHT]), and 3) training for individual finger movements (FT). ViHT and TaHT are explained in detail in a previous study [8]. The descriptions of each training program are as follows. The seven gamified training programs were intended to offer a step-by-step approach based on the progress of the participants.

ViHT consists of “placing arm” and “grasp and release.” The participants were asked to move their arms and grasp and release their hands according to the instructions provided on the monitor.

TaHT consists of “matching the same shape,” “moving the object,” and “stacking the objects.” Six different objects were used in each training session. The objects consisted of three different shapes (triangles, squares, and circles) and colors (red, blue, and green) and two sizes (large and small). Participants were asked to move a specific object to a specific area reflected on the monitor.

**Fig. 2:**
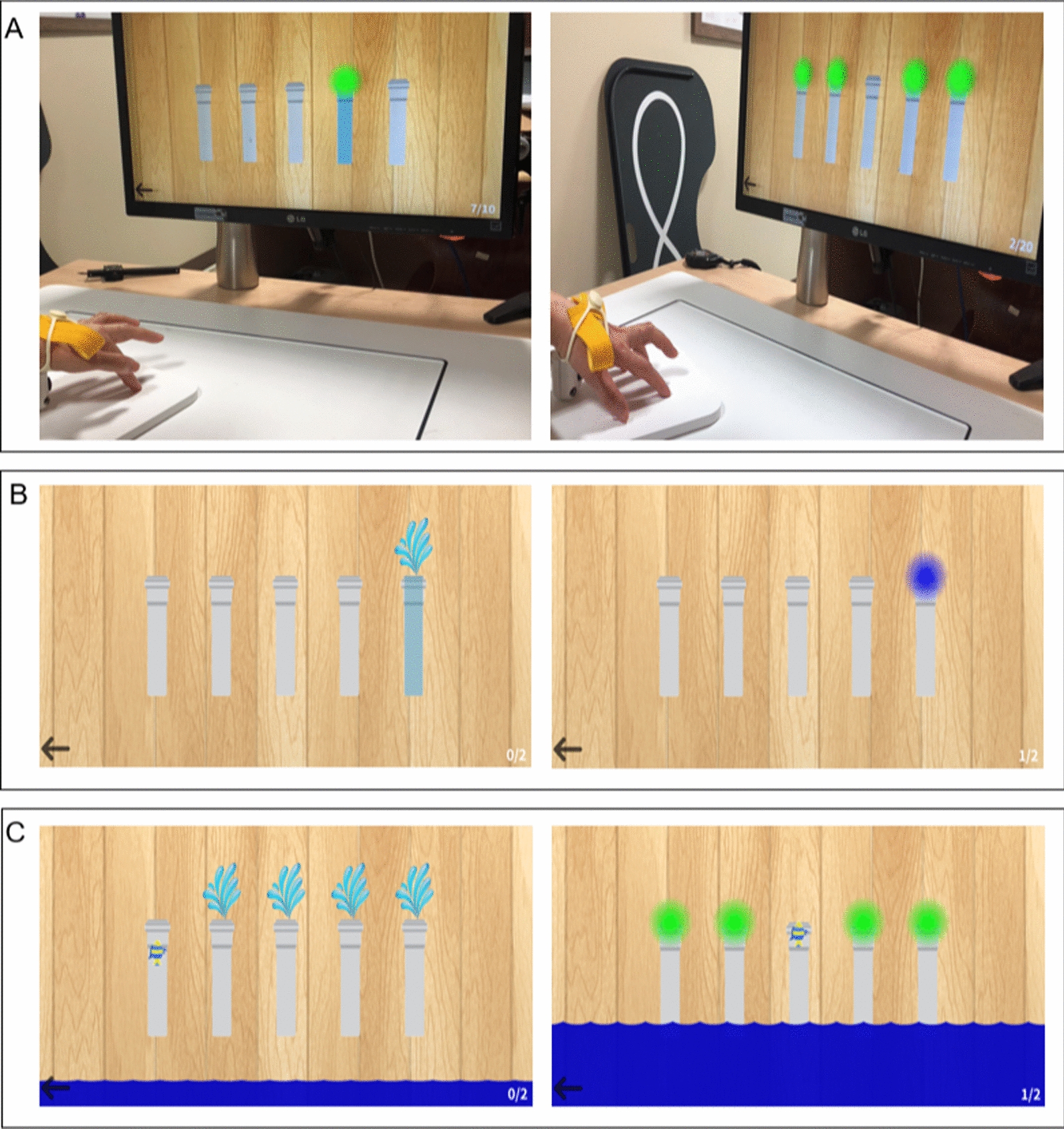
Views of the finger training and screenshots of each game. The participants sat in front of a monitor wearing TapSix and were instructed to move individual fingers according to the task. **A** Training of single- and multi-tapping tasks. **B** Screenshots of the single-tapping task. **C** Screenshots of the multi-tapping task

FT consists of the “single finger-tapping task” and “multi-finger-tapping task” (Fig. 2). Five pipes were displayed on the screen, and each pipe reflected the movement of each finger. The "single finger-tapping task" involves pressing one leaked pipe among five pipes by tapping a finger. The "multi-finger-tapping task" involves rescuing the fish by blocking the entrance of pipes with four fingers except for the pipe in which the fish was located.

### Procedures

**Fig. 3:**
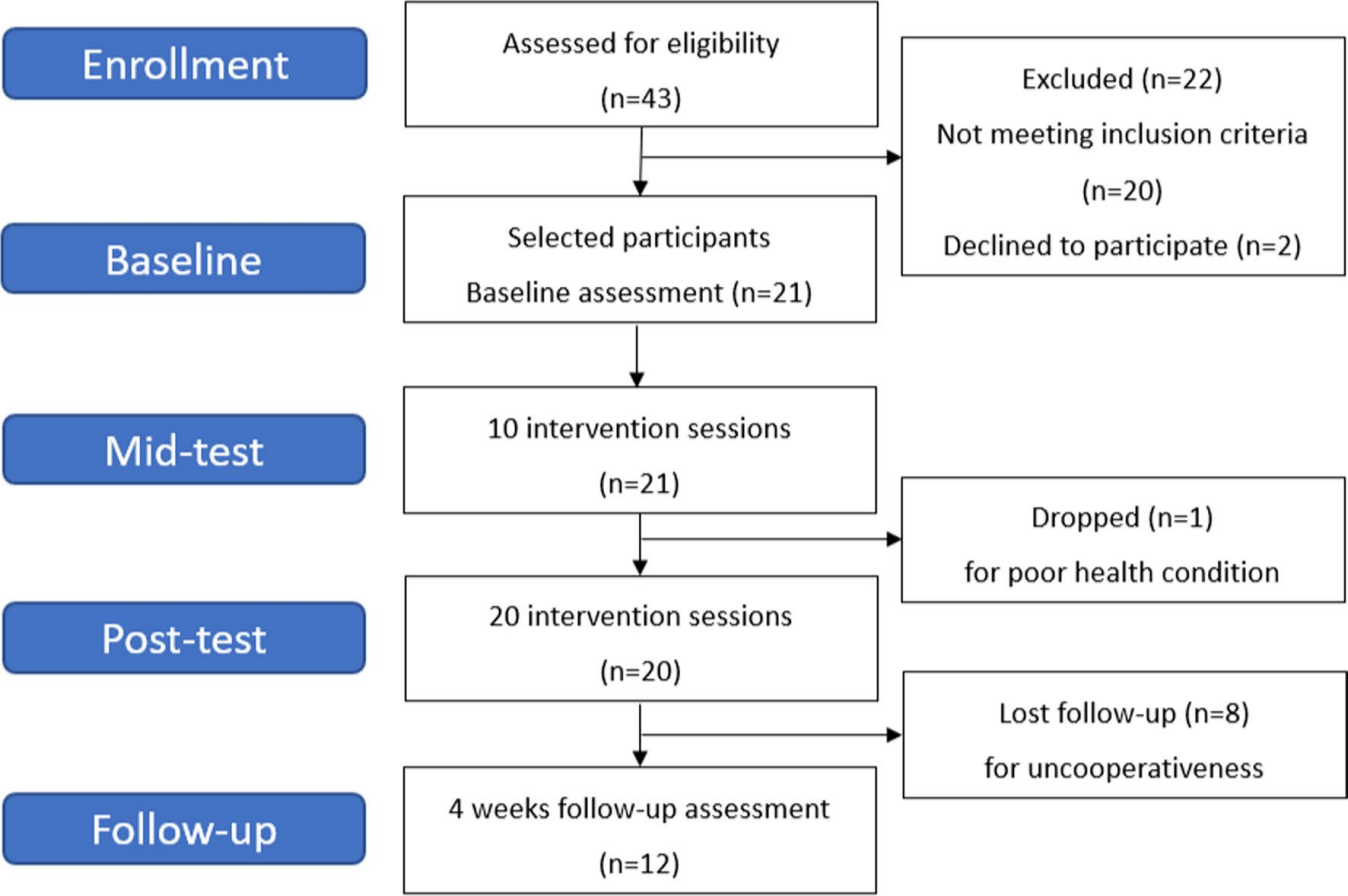
Flowchart of the clinical study

All participants sat at a desk facing a monitor and placed their hands on the MR board. During the FT, they were trained with a TapSix camera in their hypothenar. The camera supported the hand so that the FT required less strength from arms than the fingers (Fig. 2A). The participants performed 20 self-training sessions (5 days per week for 4 weeks) involving 30 min of training using MR-board 2 in a research intervention room. They did not receive any other interventions except for the MR-board 2 training. On the first MR-board-training day, an experienced occupational therapist provided brief instructions for each training program. A flow diagram of the study procedure is shown in Fig. 3.

We developed training programs and applied them according to the participants’ hand function levels based on Brunnstrom stage. In Brunnstrom stage 4 in which spasticity begins to decrease and more coordinated movement emerges, applying a rigorous home therapy program or gamified neurorehabilitation devices would facilitate the hand recovery [23, 24]. Therefore, participants with Brunnstrom stage 4 of the hand received both ViHT and TaHT, as preferably suggested, because the MR-board 2 had a special advantage for tangible user experience from MR based on our previous study [8]. In Brunnstrom stage 5 or 6, in which the combination of hand and finger movement is available, more dexterous exercise to increase fine motor movements is required [23, 24]. Participants in this stage primarily performed FT owing to the updated characteristics of MR-board 2. In summary, the participants started with ViHT and TaHT, and FT was added sequentially as the participants became accustomed to hand training and were able to move their fingers. Although it varied with each participant, most participants were recommended to exercise in the order of ViHT, TaHT, and FT in one training session. The participants exercised their upper extremities alone, following the directions of the system presented on the monitor. The participants played all seven gamified programs, ranging from a minimum of 2 min to a maximum of 7 min, an average of 4 min each. At the beginning of each training program, the participants determined the amount of each intervention and selected the program's difficulty level. The number of repetitions is displayed on the monitor, and participants can adjust the number of repetitions as needed. The therapist was in the same research room but separated from the participants using a partition. The therapist was always ready for potential safety concerns without intervening during the training and assisted the participants when they needed help.

### Outcome measures

An experienced occupational therapist assessed the outcomes. Evaluations were conducted four times: pre-training, mid-training, post-training, and follow-up (after 4 weeks of training). The data on sex, age, the affected side of paresis, and post-stroke duration were collected as demographic characteristics. We collected clinical outcome measurements for upper extremity functions as follows: Fugl–Meyer assessment (FMA) upper extremity score, repeated number of finger flexion and extension (Repeat-FE), thumb opposition test (TOT), Box and Block Test (BBT) score, Wolf Motor Function Test (WMFT) score, and Stroke Impact Scale (SIS) version 3.0. These outcomes reflect body function and structure (FMA, RF, and TOT), activity (BBT and WMFT), and participation (SIS), thus capturing the three domains indicated by the ICF [18].

The FMA is a performance-based quantitative measure for patients with stroke, with a higher score indicating a higher motor function [25, 26]. We used four outcomes of the FMA: FMA-total (33 items; score: 0–66), FMA-proximal (18 items; score: 0–36), FMA-distal (12 items; score: 0–24), and FMA-coordination (three items; score: 0–6). In addition, we obtained the data on Repeat-FE, the number of repeated finger flexions and extensions within 20 s, by requesting participants to flex and extend the affected fingers as rapidly as possible [27]. The TOT measured the opposition by the thumb to other fingers. Opposition of the thumb refers to positioning the thumb pad directly opposite the distal pad of the other fingers, enabling the grasp of both small and large objects [28]. The thumb to index finger scored 2, the middle finger scored 3, the ring finger scored 4, and the little finger scored 5.

The BBT measures gross manual dexterity by counting the number of blocks that can be moved from one compartment to another within one minute [29]. The WMFT is an upper extremity assessment tool that uses timed and functional tasks [30].The WMFT consists of 17 items: 15 functional abilities and two strength-related tasks (shoulder and grip strength). The total score on the functional ability scale (WMFT score; higher scores indicated better motor function) and the total amount of time for each item (WMFT time; shorter time indicated better performance) were obtained. We used the SIS version 3.0, a stroke-specific self-reported questionnaire, to measure the health-related quality of life (HRQoL). Among the eight SIS domains, we measured five upper limb domains: strength, hand function, physical and instrumental activities of daily living (ADL/IADL), social participation, and stroke-recovery score [31, 32]. All values were normalized between 0 and 100, with higher scores indicating a better HRQoL.

The TapSix system embedded in MR-board 2 recorded the active RoM (AROM) of fingers during FT, and 14 joints in the five fingers were analyzed: metacarpophalangeal (MCP) and interphalangeal (IP) joints of the thumb, MCP joint, proximal IP (PIP) joint, and distal IP (DIP) joint of the second, third, fourth, and fifth fingers. Setting the neutral position as 0°, finger flexion and extension were expressed as positive and negative values, respectively.

A three-dimensional (3D) model of the hand at finger flexion and extension was presented based on the first and third quartile values of finger flexion and extension of AROM on the first and last days of FT.

### Statistical analysis

One-way repeated measures analysis of variance (ANOVA) was used to compare repeatedly measured outcomes, and the following normality was confirmed. For handling missing valuables in follow-up, we used the last observation carried forward (LOCF) method. The Bonferroni correction was used for the post hoc test. The TapSix system recorded finger AROM when training FT. Because FT training was performed more frequently with time, almost twice the data was collected on the last training day (22,104) compared to the first training day (12,432). Because of this imbalanced data points, finger AROM analysis findings were analyzed using Dunnett’s test for pairwise comparisons [33]. Statistical analysis was performed using R 4.2.2 (http://www.r-project.org; R Foundation for Statistical Computing, Vienna, Austria). A p-value of < 0.05 was considered statistically significant.

## Results

**Fig. 4:**
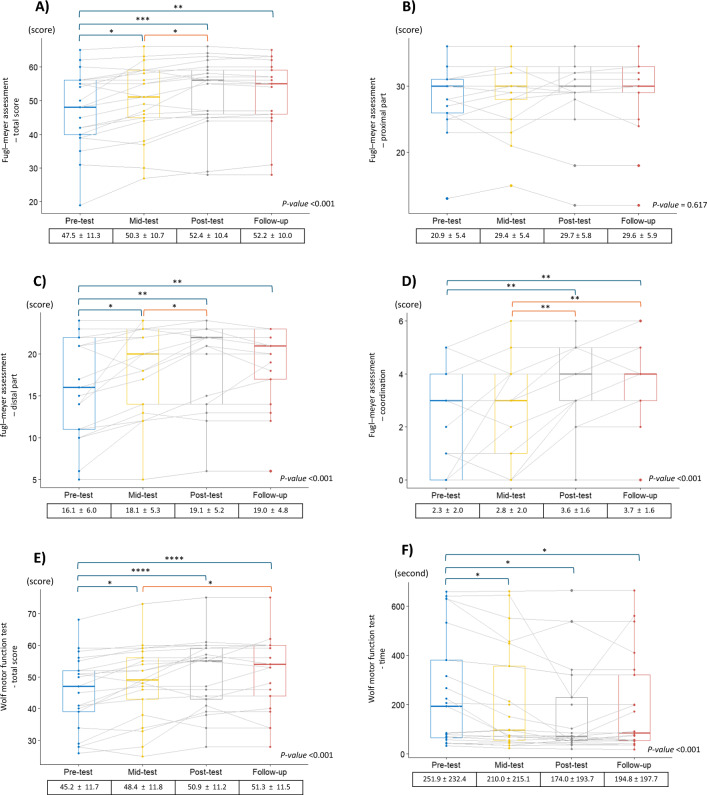

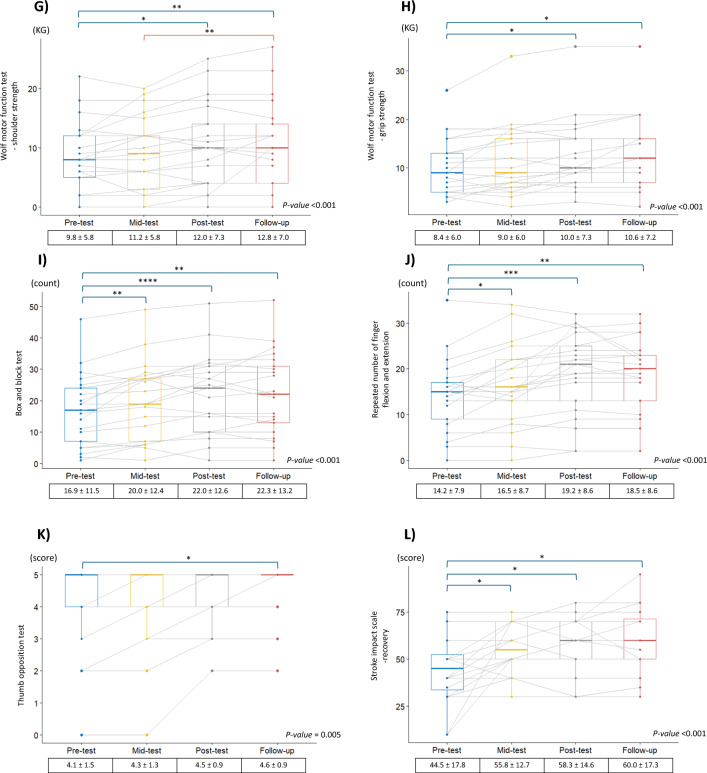
Box plots of the changes in outcome measurements and the statistical significance. The pre-, mid-, post-, and follow-up tests of the outcome measurements in boxplots. The p-values represented the results of a one-way repeated measures analysis of variance in each measurement. The Bonferroni correction results are also displayed on the boxplots according to their statistical significance. **A** Fugl–Meyer Assessment—total score. **B** Fugl–Meyer Assessment—proximal. **C** Fugl–Meyer Assessment—distal. **D** Fugl–Meyer Assessment—coordination. **E** Wolf Motor Function Test—score. **F** Wolf Motor Function Test—time. **G** Wolf Motor Function Test—shoulder strength. **H** Wolf Motor Function Test—grip strength. **I** Box and Block Test. **J** Repeated number of finger flexion and extension. **K** Thumb opposition test. **L** Stroke Impact Scale—recovery. *Annotation.* Statistical significance indicated as < 0.05*, < 0.01**, < 0.001***, and < 0.0001****

Of the 21 participants, one dropped out during intervention due to pneumonia, irrelevant to this study. Figure 4 presents the box plots of each outcome measurement. In addition, the supplementary table indicates the results of subgroup analysis by Brunnstrom stage according to ICF domains. Based on one-way repeated-measures ANOVA and its post hoc test, except for the FMA-proximal scores, other FMA, Repeat-FE, TOT, BBT, and WMFT values improved significantly following MR-board-2 training as they underwent the pre-, mid-, and post-tests. Post hoc analysis demonstrated that the variables improved throughout the MR-board-2 training and did not change from post-test to follow-up, indicating that these variables were maintained after training until follow-up. SIS-stroke recovery was improved throughout training and follow-up (*p* < 0.001).

**Fig. 5:**
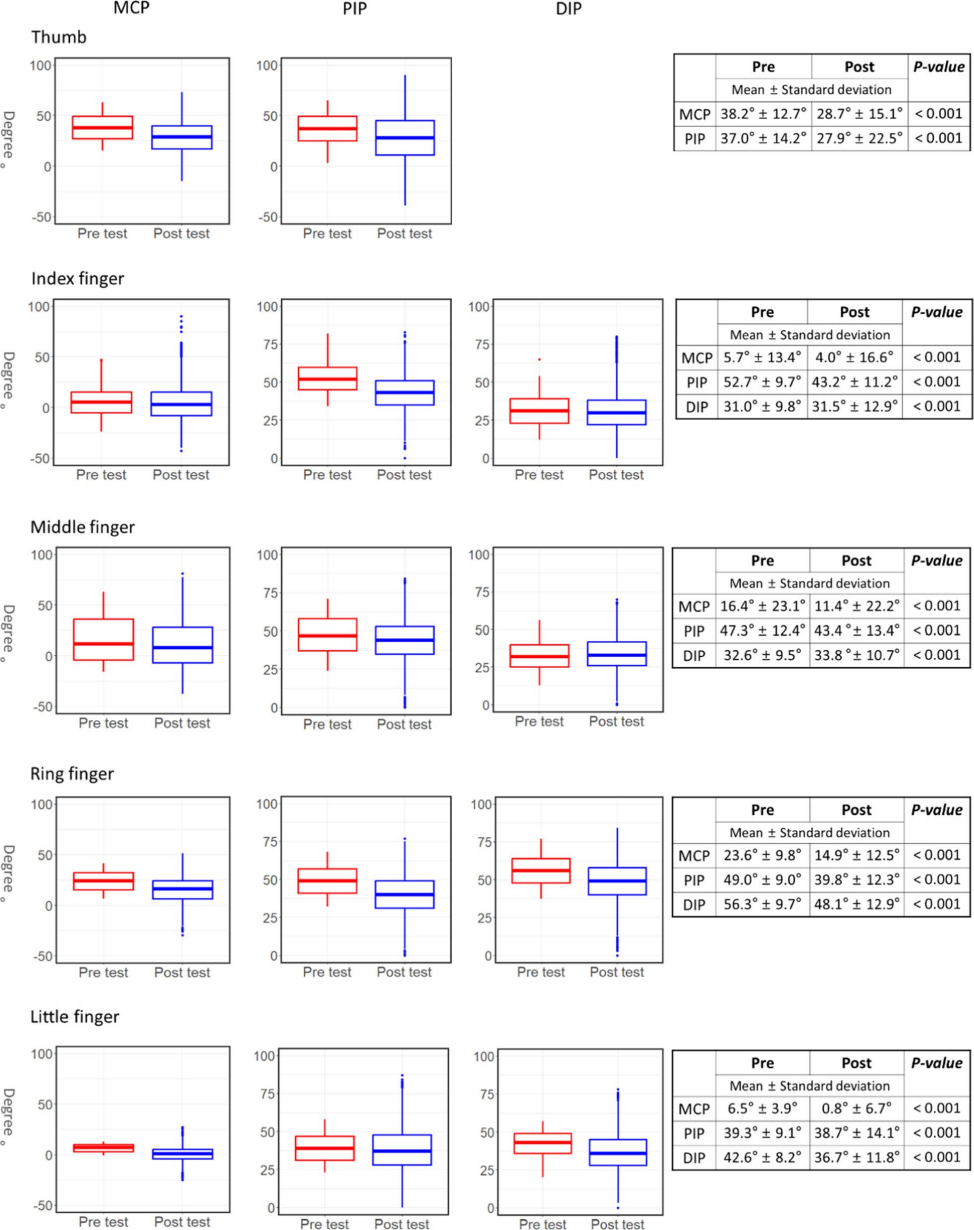
Box plots of the range of motion of each finger joint. Data are presented as mean ± standard deviation. Dunnett’s test was applied for pairwise comparisons of pre- and post-test. *MCP* metacarpophalangeal joint, *IP* interphalangeal joint, *PIP* proximal IP, *DIP* distal IP. Boxplot’s upper and lower whiskers displayed 95% confidence interval, which indicated the range of finger movement. Mean and median of finger range indicated the finger

**Fig. 6:**
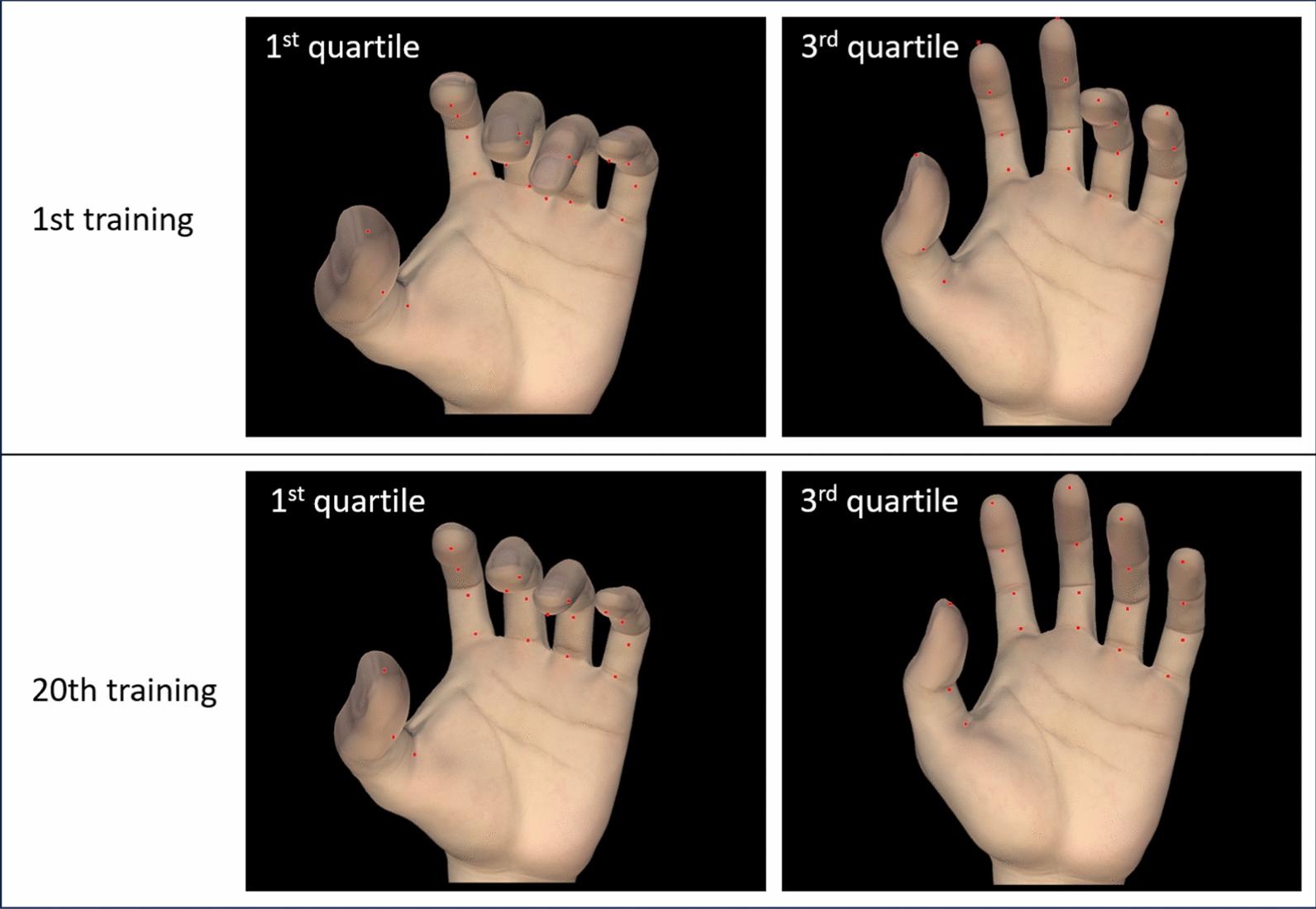
Three-dimensional hand model during finger training. These images were prepared based on the whole range of motion data from the individual fingers of participants

We collected finger movement data on the first and 20th days of FT; 12,432 and 22,104 data points were obtained on the first and 20th training days, respectively, indicating an increase in FT time. Figure 5 displays finger movements in the box plot, in which we found that the upper and lower whiskers became wider on the 20th day compared with the first day. The broader range of confidence intervals indicated that the finger’s AROM was increased. The mean of finger AROM was shifted toward less flexion and more neutral position (toward 0°) in finger joints except for the DIP joints of the index and middle fingers. After the Dunnett test, all AROM values of finger joints significantly changed throughout the MR-board intervention (*p* < 0.001). Figure 6 depicts the 3D hand reconstruction based on the first and third quartile data of AROM values of each joint.

## Discussion

This study demonstrated that MR-board-2 self-training, an MR-based rehabilitation program involving FT, significantly improved upper limb functions in terms of impairment level (FMA, RF, and TOT), activity (BBT and WMFT), and participation (stroke recovery item of SIS). Moreover, these effects were maintained for 4 weeks after training when statistical techniques were applied to handle missing data (nine out of 20 missed follow-up). Also, individual finger AROM values showed improvements. The subtest results of the FMA indicated that the improvement in the upper extremity primarily occurred in the distal part rather than the proximal part after the MR-board-2.0 self-training. The discrepancy in the results, where improvement was seen in FMA distal but not in FMA proximal, indicates the task specificity of MR-board 2. This might be due to the characteristics of TaHT and FT, which involve fine motor-related hand movements, including manipulating objects and finger-individuation movements. Substantial evidence supports the effectiveness of task-specific training as a neuromotor intervention in neurological rehabilitation [34]. The results indicate that MR-board-2 self-training in participants who were in the chronic phase of stroke and did not receive interventions other than MR-board-2 training resulted in functional improvements across a variety of domains. Additionally, participants expressed high satisfaction with the intervention as they perceived improvement after using the MR board, based on their SIS-recovery scores. However, we were unable to link the effectiveness of the MR board to participation in the ICF model as the remaining SIS scores were not significant. Longer-term projects are required to elucidate the positive effects associated with participation.

The effects of the MR-board-2 self-training became more evident than those of the original MR-board training used in a previous study [8], possibly because the MR-board-2 training included FT, enabling more complex training for an extended duration. The FT in MR board improved multi-finger capacity, which is composed of finger strength and individuation [11]. Individuation is a crucial independent movement of the digits in ADLs; relatively few studies have assessed the impact of explicitly targeting individuated movement on hand rehabilitation [9]. Therefore, TOT, which represents finger individuation improved with MR-board-2 training.

Studies have been conducted recently to collect kinematic data on finger individuation using censored gloves or 3D-motion capture in healthy adults [35, 36]. Similarly, MR-board 2 also collected kinematic data of FT executed by TapSix, a camera-based computer vision technology; in contrast, most FT programs in virtual reality rehabilitation commonly use wearable glove-type devices [37–39]. Participants easily wore TapSix with a strap on their hypothenar area, allowing more stable imaging without restricting wrist motion and not wearing gloves on individual fingers. All participants in the present study could wear the TapSix by themselves. TapSix was robust under various lighting conditions [17]. Owing to the proximity of the camera to the fingers, the lightness values of the fingers were significantly higher than the hue and saturation values. Furthermore, TapSix uses a 940-nm infrared light-emitting diode (and filter), which is a convenient system for noise processing. These features enable TapSix to extract finger data by distinguishing the finger from the surface. TapSix can detect subtle finger movements using position and does not require specific movements, such as contacting sensing pads between fingers [40]. Additionally, the ViHT provided haptic feedback to boost motor learning [41], which was impossible in training using a glove. Sensory feedback from tangible objects during TaHT and various surfaces during FT enables the experience of a realistic sense of touch and proprioception in MR, leading to motor control enhancement.

Based on the kinematic data from TapSix, we found the increased AROM in each finger, and the participants moved their fingers more frequently after training. In addition, hand posture was normalized more successfully in the last training session than in the first one, indicating that finger movements of the participants became more natural after FT. The mean of all joint-ROM values became less flexed after the training, except for the index and middle finger DIP joints of the 14 finger joints. This observation might be attributed to the fact that the posture became more relaxed and natural throughout the training, and the DIP joints in the index and middle fingers, critical for hand manipulation, played more active and focused roles [42].

This study has limitations. First, this study was not a randomized controlled trial; thus, it was not sufficient to confirm the effects of MR-board-2 training. However, our findings could indicate the feasibility of MR-board 2 because the study was conducted among participants with chronic stroke without other interventions. In addition, four tests and follow-up observations confirmed the effects of MR-board-2 training, related to improvement during the intervention and maintenance of the scores until the follow-up test. Second, the number of participants was small with a relatively high drop-out rate in follow-up examination. The low participation in follow-up examination might be because the participants did not have any merit for participating in the outcome assessment such as financial or therapeutic benefit. In future studies, measures should be taken to minimize the drop-out rate during the follow-up test phase. Third, the components and amount of specific training in MR-board 2 were variable among the participants because we only recommended the training structure, such as the order of training or adjustment difficulty, making it difficult to compare the effects of specific training. However, because MR-board 2 was used as a tool for in-home rehabilitation, these variations could be understood as a reflection of the participants' free will and training at home. Finally, we did not use standardized measurement tools for finger individuation. We used the TOT to check individuation, which is not standardized and mainly for thumb motion. In addition, the kinematic data could not demonstrate finger individuation.

## Conclusions

MR-board 2 could provide participants with immersive natural interaction between humans and computers via haptic somatosensory and visuospatial interactions. The convergence of different technologies on MR-board 2 enables effective rehabilitation, resulting in functional improvements in patients with stroke. MR-board 2 contains gamified finger- and hand-training programs, allowing effective repetitive movements. It is capable of recording and assessing performance and immediate feedback, enabling self-training without continuous supervision from a healthcare provider, and has no adverse effects, such as falls or pain. These features warrant MR-board 2 as a self-training tool that significantly improved the upper limb functions reflected by the impairment level (FMA, RF, and TOT), activity (BBT and WMFT), and participation (stroke recovery item of SIS) based on the ICF model among people with stroke. The findings of this study provide a new approach of rehabilitation for patients with stroke.

### Supplementary Information

Supplementary Material 1.

## Data Availability

The datasets used and/or analysed during the current study are available from the corresponding author on reasonable request.

## Abbreviations

MR: Mixed reality
MRR: Mixed reality-based rehabilitation
IMU: Inertial measurement unit
FT: Finger training
ICF: International Classification of Functioning, Disability, and Health
MAS: Modified Ashworth scale
RoM: Range of motion
ViHT: Virtual hand training
TaHT: Tangible hand training
FMA: Fugl–Meyer assessment
Repeat-FE: Repeated number of finger flexion and extension
BBT: Box and Block Test
WMFT: Wolf Motor Function Test
SIS: Stroke Impact Scale
HRQoL: Health-related quality of life
ADLs: Activities of daily living
IADLs: Instrumental ADLs
AROM: Active RoM
MCP: Metacarpophalangeal
IP: Interphalangeal
PIP: Proximal IP
DIP: Distal IP
ANOVA: Analysis of variance
