# Supplementary material for: Effectiveness of mixed reality-based rehabilitation on hands and fingers by individual finger-movement tracking in patients with stroke
Source: J Neuroeng Rehabil. 2024 Aug 10;21:140. doi: 10.1186/s12984-024-01418-6 (PMC11316335; doi:10.1186/s12984-024-01418-6)
Supplement: Supplementary file 1 — Supplementary Material 1. [file 12984_2024_1418_MOESM1_ESM.docx]

**Supplementary table. Results of outcome measurements and the subgroup test of MR-board-2 training via the ICF model**

| Outcome measurements |  | Pre-test | Mid-test | Post-test | Follow-up | *p-*value |
| --- | --- | --- | --- | --- | --- | --- |
| **Body function and structure** | | | | | | |
| FMA-proximal | BS 4 | 25.8 ± 5.3 | 25.9 ± 5.3 | 26.4 ± 6.5 | 26 ± 6.5 | 0.912 |
|  | BS 5 and 6 | 32 ± 3.6 | 32.6 ± 3.1 | 32.6 ± 2.9 | 32.8 ± 2.8 | 0.390 |
| FMA-distal | BS 4 | 11.9 ± 5.1 | 14.2 ± 4.8 | 15.2 ± 5.1 | 15.6 ± 4.9 | 0.003 |
|  | BS 5 and 6 | 20 ± 3.8 | 21.7 ± 2.4 | 22.7 ± 1.1 | 22.1 ± 1.6 | 0.054 |
| FMA-coordination | BS 4 | 1.8 ± 2.2 | 2.1 ± 2.2 | 2.8 ± 1.9 | 3 ± 1.9 | 0.030 |
|  | BS 5 and 6 | 2.7 ± 1.7 | 3.4 ± 1.6 | 4.3 ± 1.0 | 4.3 ± 1.0 | <0.001 |
| FMA-total | BS 4 | 39.5 ± 9.8 | 42.2 ± 9.1 | 44.4 ± 9.6 | 44.6 ± 9.1 | 0.003 |
|  | BS 5 and 6 | 54.7 ± 6.9 | 57.7 ± 5.4 | 59.6 ± 3.7 | 59.2 ± 3.7 | 0.011 |
| Repeat-FE | BS 4 | 10.8 ± 7.1 | 12.5 ± 8.3 | 14.6 ± 9.5 | 13.7 ± 8.3 | 0.002 |
|  | BS 5 and 6 | 17.4 ± 7.6 | 20.2 ± 7.7 | 23.4 ± 6.3 | 22.9 ± 6.4 | 0.002 |
| TOT | BS 4 | 3.5 ± 1.8 | 3.7 ± 1.7 | 4.1 ± 1.1 | 4.2 ± 1.1 | 0.033 |
|  | BS 5 and 6 | 4.6 ± 0.7 | 4.8 ± 0.4 | 4.9 ± 0.3 | 4.9 ± 0.3 | 0.057 |
| **Activity** |  |  |  |  |  |  |
| BBT | BS 4 | 7.7 ± 5.6 | 9.5 ± 6.1 | 11.8 ± 7.2 | 11.1 ± 6.1 | 0.029 |
|  | BS 5 and 6 | 25.2 ± 8.6 | 29.5 ± 8.0 | 31.4 ± 8.4 | 32.5 ± 8.4 | <0.001 |
| WMFT score | BS 4 | 35.8 ± 8.2 | 40.0 ± 10.0 | 42.8 ± 9.3 | 42.9 ± 10.0 | <0.001 |
|  | BS 5 and 6 | 53.8 ± 6.4 | 56.1 ± 7.1 | 58.3 ± 6.7 | 58.9 ± 6.2 | <0.001 |
| WMFT time (s) | BS 4 | 435 ± 210 | 378 ± 206 | 308 ± 213 | 340 ± 203 | 0.005 |
|  | BS 5 and 6 | 85.3 ± 63.5 | 57.0 ± 21.6 | 52.5 ± 15.9 | 62.9 ± 41.2 | 0.188 |
| WMFT shoulder strength | BS 4 | 8.3 ± 5.0 | 8.9 ± 5.3 | 9.8 ± 4.8 | 10.2 ± 5.4 | 0.032 |
|  | BS 5 and 6 | 11.1 ± 6.3 | 13.3 ± 8.3 | 14.1 ± 8.8 | 15.1 ± 8.8 | 0.001 |
| WMFT grip strength | BS 4 | 5.6 ± 4.3 | 6.8 ± 5.5 | 7.0 ± 6.0 | 7.6 ± 5.8 | 0.116 |
|  | BS 5 and 6 | 10.9 ± 6.4 | 10.9 ± 6.8 | 12.8 ± 7.4 | 13.3 ± 7.5 | 0.027 |
| **Participation** |  |  |  |  |  |  |
| SIS-strength | BS 4 | 38.2 ± 14.5 | 35.1 ± 6.2 | 32.0 ± 10.1 | 36.1 ± 15.9 | 0.568 |
|  | BS 5 and 6 | 30.7 ± 16.6 | 38.7 ± 16.7 | 38.1 ± 16.4 | 39.8 ± 17.3 | 0.021 |
| SIS-hand function | BS 4 | 22.8 ± 24.9 | 16.7 ± 25.6 | 17.8 ± 21.8 | 17.8 ± 25.0 | 0.011 |
|  | BS 5 and 6 | 47.7 ± 21.7 | 51.8 ± 21.0 | 58.6 ± 20.5 | 54.1 ± 21.7 | 0.072 |
| SIS-ADL/IADL | BS 4 | 70.6 ± 14.7 | 70.3 ± 14.2 | 69.7 ± 12.2 | 73.3 ± 11.1 | 0.499 |
|  | BS 5 and 6 | 82.3 ± 19.7 | 76.6 ± 18.2 | 78.6 ± 18.7 | 78.9 ± 18.3 | 0.313 |
| SIS-social participation | BS 4 | 40.6 ± 27.2 | 46.2 ± 28.1 | 46.9 ± 30.4 | 47.2 ± 25.2 | 0.572 |
|  | BS 5 and 6 | 51.7 ± 17.0 | 45.1 ± 23.9 | 50.8 ± 23.2 | 58.8 ± 19.1 | 0.233 |
| SIS-stroke recovery | BS 4 | 42.2 ± 17.9 | 53.3 ± 15.0 | 54.4 ± 15.1 | 53.3 ± 15.0 | 0.152 |
|  | BS 5 and 6 | 46.4 ± 18.5 | 57.7 ± 10.8 | 61.4 ± 14.2 | 65.5 ± 17.8 | <0.001 |

FMA, Fugl–Meyer assessment; Repeat-FE, repeated number of finger flexion and extension; TOT, thumb opposition test; BBT, Box and Block Test; WMFT, Wolf Motor Function Test; BS, Brunnstrom stage; MR, mixed reality; ICF, International Classification of Functioning, Disability, and Health; SIS, Stroke Impact Scale; ADLs, activities of daily living; IADLs, instrumental ADLs.

†One-way repeated-measures analysis of variance for comparison of changes

‡Data are presented as mean ± standard deviation.
